# BIOMOLECULAR ACTIVITY OF CRYPTOCOCCUS DURING CRYPTOCOCCOSIS: A REVIEW OF MOLECULAR INTERACTIONS OF CRYPTOCOCCUS WITH HUMAN IMMUNE SYSTEM AND BLOOD-BRAIN-BARRIER

**DOI:** 10.21010/Ajidv18i1.3

**Published:** 2023-10-20

**Authors:** JULIAN Julian, ADAWIYAH Robiatul, WAHDINI Sri

**Affiliations:** 1 Master’s Programme in biomedical science, Faculty of Medicine, Universitas Indonesia, Jakarta, Indonesia; 2 Department of Parasitology, Faculty of Medicine, Universitas Indonesia, Jakarta, Indonesia

**Keywords:** Cryptococcus, Biomolecular Activity, Immune Response, Fungal Disease, Blood-Brain-Barrier

## Abstract

Global mycosis is still a problem. One of these is the cryptococcal disease. A systemic mycosis brought on by Cryptococcus is called cryptococcosis. Host immunological conditions influence infection with Cryptococcosis. When environmental spores are inhaled by the host, the spores get to the lungs, an infection is created. Alveolar macrophages and other immune cells recognize *Cryptococcus* in the lung. The initial line of defense against pathogens in the phagolysosome is provided by alveolar macrophages found in the lungs. When the immune system is weak, Cryptococcus uses the evasion system as a molecular interaction with the immune system and persists in the lungs without causing any symptoms such as Factor Transcription, Cell masking, N-glycan structure, Extracellular molecule, and Antioxidant system. The evasion mechanism protects and makes Cryptococcus disseminate throughout the other organs, especially CNS. If Cryptococcus escapes against the host immune system, it will disseminate to other organs, especially Cerebrospinal System by Three mechanisms. There are Trojan Horse, Paracellular, and Transcellular interactions with Blood-Brain Barrier. Disease severity is determined by the Interaction between the host’s immune system and the fungus.

## Introduction

Mycosis, otherwise known as cryptococcosis, is caused by *Cryptococcus spp*. and it affects a variety of organs, including the lungs, skin, bones, and brain (Zhao et al., 2023). In 1894, Francisco San Felice discovered the first strain of Cryptococcus. In the same year, two German surgeons, Otto Busse, and Abraham Buschke reported a case of cryptococcosis in the infected tibia. A case of cryptococcosis in the CNS was documented in 1912 by Rusk and Farnell (Busse, 1894; Departemen Parasitologi, 2008) In 2020, WHO observed 179,000 cases of cryptococcal antigenemia, 152,000 cases of cryptococcal meningitis, and 112,000 mortality (World Health Organization, 2022). Cryptococcosis infection is becoming more common in immunocompromised persons, particularly those who are HIV-positive. In 16–30% of HIV-positive patients in Indonesia, cryptococcosis was associated with abnormalities of the CNS (Adawiyah, Rozaliyani and Wahyuningsih, 2019).

*Cryptococcus neoformans* is the principal causative agent of most Cryptococcal infections (Sjamsuridzal and Wahyuningsih, 2016; Diniz-Lima et al., 2022). Alveolar macrophages will phagocytose basidiospores when they are breathed into the lung alveoli. Infections with Cryptococcus are uncommon in individuals with healthy immune systems, but in those with weakened immune systems, Cryptococcus can use the evasion system to suppress immune responses like the protein extracellular, small molecule, N-glycan structure, antioxidant system, and the role of Transcription factor to evade killing mechanisms and avoid clearance. The fungus will spread to other organs if it manages to evade the immune reaction. If Cryptococcus evades the host immune system, it will disseminate to other organs, especially the CNS. Few details on the immunological reaction of the host and all of the Cryptococcus biomolecular response have been revealed. In this paper, we will review about biomolecular activity of Cryptococcus, especially molecular interaction against the immune system and Blood Brain Barrier.

### Overview of Human Immune System against Cryptococcus Infection

### Recognition mechanism

Infection begins when the host inhales basidiospores which are relatively dehydrated yeast cells, Because of their small size basidiospores can enter alveoli in the lungs and against innate immune response, like alveolar macrophages and Dendritic Cells (DCs). The antigen is recognized antigens as Pathogen-associated molecular patterns (PAMP) by Pattern Recognition Receptors (PRRs) (Sjamsuridzal and Wahyuningsih, 2016; Campuzano and Wormley, 2018; Sato and Kawakami, 2022). Three major sub-families of PRRs can recognize Cryptococcus neoformans as follow:

### Toll-Like Receptors (TLRs)

TLRs act as functional receptor proteins to identify pathogens and activate the innate immune systems. TLR-2, TLR-4, and TLR-9 are the three TLR subtypes that can identify Cryptococcus as an antigen. TLR-2 can recognize the -glucans in the cell walls of Cryptococcus, but the polysaccharide capsule, which contains GXM and GalXM, prevents Cryptococcus from being recognized, causing the polysaccharide capsule to be recognized by TLR-4. TLR9 is yet another TLR subtype that can identify Cryptococcus. The unmethylated CpG region in the DNA of Cryptococcus will be recognized by TLR9. TLRs have a TIR domain at their C-terminus and an LRR at their N-terminus. Recognition of PAMP by TLR initiates signal transduction and induces transcription of various inflammatory genes to clearance pathogens (Arthur and Ley, 2013; Murphy and Weaver, 2016; Campuzano and Wormley, 2018; Ma’at, 2018; Dobashi-Okuyama et al., 2020; da Silva-Junior et al., 2021; Onyishi et al., 2023).

### C-Type Lectin Receptors (CLRs)

CLRs are proteins that are expressed by myeloid cells, including DCs and macrophages (Walsh et al., 2017). CLRs are divided into three groups to identify Cryptococcus: Mannose Receptors, Dectin-2, and DC-SIGN. The mannose receptor, also known as CD206, participates in phagocytosis, notably in preparing and presenting antigens to T cells from naïve lymphocytes. Terminal mannose residues identify Cryptococcus, although intracellular mannose receptor signaling is unclear (Campuzano and Wormley, 2018; Höft, Hoving and Brown, 2020). DC-SIGN as known as CD209 binds mannose residues and fucose and induces a signal transduction cascade to express genes of pro-inflammatory cytokines and involve antigen uptake (Heung, 2017). Dectin-2 can recognize mannose and α-mannan to eradicate and clear *Cryptococcus neoformans*, Dectin-2’s recognition of Cryptococcus causes a mechanism of macrophage activation, DC maturation, the formation of Reactive Oxygen Species (ROS), and the expression of proinflammatory cytokines (Roth et al., 2016; Tang et al., 2018).

### NOD-Like Receptors (NLRs)

Cytoplasmic receptors called NLRs recognize DAMPs (damage-associated molecular patterns) and release IL-1β. Monocyte, macrophage, and neutrophil recruitment are aided by IL-1β, which also improves phagocytosis by raising reactive oxygen species (ROS) and nitric oxide synthase (NOS) and leads to the activation of Th1 and Th17 (Guo, C. et al., 2014; Abbas, Lichtman and Pillai, 2019; Williams, Gonzales-huerta and Armstrong-james, 2021).

### Another receptor: Macrophage Receptor with Collagenous Structure (MARCO)

MARCO also referred to as scavenger receptors, can attract phagocytic mononuclear cells to the site of the infection, increase the secretion of pro-inflammatory cytokines, and move dendritic Cells. But the signaling route is not identified (Campuzano and Wormley, 2018).

### Mechanism of Human Immune Response against Cryptococcus

**Figure 1:**
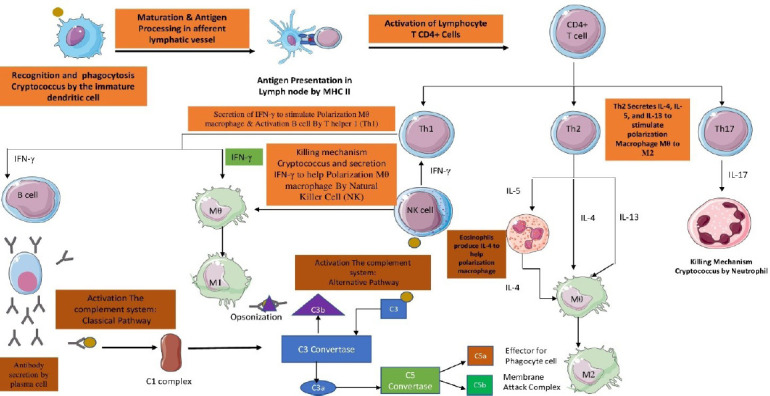
Host Immune Response. Parts of the figure were drawn using pictures from Servier Medical Art. Servier Medical Art by Servier is licensed under a Creative Commons Attribution 3.0 Unported License (https://creativecommons.org/licenses/by/3.0/)

Cryptococcus enters the human body through inhalation and is first encountered by macrophages (Campuzano and Wormley, 2018). On the other hand, CD4+ T cells are activated by DCs presenting antigens to naive T cells. Th1, Th2, and Th17 T cells can develop from CD4+ T cells. Th1, Th2, and Th17 cells release cytokines that polarize macrophages as the first line of defense. Macrophages are the initial line of defense against many common pathogens and are cellular innate immunity. Alveolar macrophages are one type of macrophage. Using PRRs, macrophages can identify antigens and polarize into classically activated (M1) and alternatively activated (M2) cells. The polarization of macrophages during infection is determined by the dominating cytokine environment (Davis et al., 2013; Garelnabi and May, 2018). M1 macrophages are polarized by cytokines secreted by other immune cells, like IFN -γ by Th1 (Leopold Wager and Wormley, 2015; Punt et al., 2019). M1 macrophages produce NOS through reactions between iNOS enzymes that work with L-arginine substrates to produce Nitric oxide (NOS) which acts as an antifungal (Fu et al., 2018; Herb and Schramm, 2021). Meanwhile, M2 macrophages become polarized when IL-4 and IL-13 become more dominant in the cytokine environment, which has roles in the repair mechanism, the promotion of the killing mechanism, and encapsulation (Campuzano and Wormley, 2018). Another role of cytokine secretion by T helper activates in other immune cells like eosinophils to secrete IL-4 and modulate polarization of macrophage, and neutrophils to eliminate Cryptococcus. The other cells which contribute to eliminate Cryptococcus are NK cells. NK cells will eliminate Cryptococcus in their killing mechanism, but NK cells secrete IFN -γ as Another source to induce B cells and Th1 (Rohatgi and Pirofski, 2017; Schmidt, Tramsen and Lehrnbecher, 2017; Li et al., 2018). IFN -γ induces B cells to produce antibodies that can associate with complement for opsonization via the traditional pathway and alternative pathway (Leichner and Kambayashi, 2014; Elsegeiny, Marr and Williamson, 2018; Kumar, Connors and Farber, 2018). These biochemical processes can produce C3 convertase, which splits C3 into C3a and C3b. A C5 convertase complex can be produced by C3a and C3b. C5 is split into two molecules, C5a, and C5b, by the C5 convertase complex. Another mechanism for activating C3 results in the formation of MAC, which lyses the fungus. In the killing mechanism of the cryptococcus, C3b is necessary for opsonization, while C5b starts the creation of the MAC. In the meantime, C3a and C5a mediate the inflammatory response and draw phagocytic effector cells (Voelz and May, 2010; Mukaremera and Nielsen, 2017; Gressler AE et al., 2021). The mechanism of action is demonstrated in Figure 1.

### Molecular Interaction of Cryptococcus Infection and Immune Response

The powerful immune system can eliminate pathogens like Cryptococcus. However, pathogens have a biomolecular response to evade the host’s immune response. Biomolecular responses are as follows:

### Transcription Factor

Gene expression results in the creation of proteins. Transcription is a prerequisite for gene expression and is triggered by transcription factors. In the cell nucleus, transcription factors are a collection of proteins that help translate the genetic code into mRNA. Functional proteins that are involved in the immune response’s survival and evasion mechanisms are activated by transcription factors during a Cryptococcus infection. In Cryptococcus, the following transcription factors exist:

**Table 1:** Some Transcription Factors and Their Functions

No	Transcription Factor	Function	References
**1**	**Pdr802**	Pathogenicity of Cryptococcus and Control of Titan Cell Formation	(Reuwsaat *et al*., 2021
		Modulation of secretion enzyme: Urease, catalase, chitin deacetylase	
**2**	Liv4	Growth and virulence factor	(Yi *et al*., 2020)
**3**	Gat204	maintain anti-phagocytic activity	(Chun, Brown and Madhani, 2011)
**4**	Blp1		
**5**	Rim101	Regulation of the host-pathogen interaction and cell wall composition	(O'Meara *et al*., 2014)
**6**	Bzp4,	Biosynthesis of melanin	(Yang *et al*., 2022)
**7**	Usv101		
**8**	Hob101		
**9**	Mbs1		
**10**	cuf1	Regulator of SOD1 and SOD2 superoxide dismutases production	(Aaron D Smith *et al*., 2021)
**11**	Crz1/Sp1	keeping the cell wall and plasma membrane stable	(Lev *et al*., 2012)
**12**	Usv101	Polysaccharide shedding and capsule thickness regulation	(Dambuza, I.M. *et al*., 2018)
**13**	AP-1	Redox sensor YAP1 regulation in Cryptococcus oxidative stress responses	(So *et al*., 2019)
**14**	APSES-like transcription factor, Mbs1 (Mbp1- and Swi4-like protein 1)	Antifungal drug resistance, stress response, and virulence of Cryptococcus neoformans: pleiotropic roles	(Song *et al*., 2012)
**15.**	Gti1/Pac2	Regulation morphological transition and virulence, mating, and secondary metabolism	(Paes *et al*., 2018)
**16**	Hcm1	Stress oxidative protection	(Ke *et al*., 2022)
**17**	ZnF2	a regulator of morphogenesis that controls the yeast-to-hyphal transition in Cryptococcus	(Lin *et al*., 2010; Lin, Idnurm and Lin, 2015)

### Cell Masking

Cryptococcus polysaccharide capsule is the primary pathogenicity factor located externally to the cell wall. Based on biochemical structure, 88% glucuronoxylomannan (GXM), 10% galactoxylomannan (GalXM), and 2% mannoproteins are found in the *Cryptococcus neoformans* capsul (Casadevall, Coelho and Alanio, 2018; Decote-Ricardo et al., 2019) The Capsule utility in the evasion system can prevent phagocytosis by preventing the digestion of phagosomes, when the endocytosed stage, This fungus releases the polysaccharide capsule which contains GXM into the vesicles that surround the phagosome and accumulates in the cell cytoplasm, resulting in dysfunction and lysis of macrophage. This fungus can produce a big polysaccharide capsule of up to 70-100µm, which makes it difficult for this fungus to be phagocytosed (Casadevall, Coelho and Alanio, 2018; Zaragoza, 2019; Wear et al., 2022) The ex-vivo research was used to demonstrate the immunomodulatory features of the cryptococcal capsule in HIV-related cryptococcal meningitis. Enlargement of the cryptococcal capsule size is associated with decreased fungal clearance, loss of CSF leukocytes, and proinflammatory cytokines like IFN-γ (Robertson et al., 2014) GXM can inhibit antigen-presenting mechanism through induction of IL-10 secretion. The release of IL-10 reduces T-cell proliferation and MHC-II expression. GXM activates the expression of the Fas ligand or the caspase-independent pathway through the synthesis of iNOS and NO, which causes cells like macrophages and T cells to undergo apoptosis (Monari et al., 2005; Chiapello, L.S et al., 2008; Villena et al., 2008; Decote-Ricardo et al., 2019).

Recent research has shown that thermotolerance in *Cryptococcus neoformans* is linked to antigen masking via mRNA decay-dependent reprogramming. Ccr4 is a protein in *Cryptococcus neoformans* that modulates host cell conditions, including thermotolerance. The Ccr4 mutant strain increased exposure to cell wall glucans, and recognition by Dectin-1 resulted in increased phagocytosis by lung macrophages compared to the wild type, implying that mRNA decay plays a role in conditions and immune evasion (Bloom et al., 2019).

### N-glycan Structure

After protein production, which takes place in the endoplasmic reticulum, proteins can be modified enzymatically by covalent bonding. Processes like acetylation, glycosylation, and ubiquitination are examples of post-translational modification (Ramazi and Zahiri, 2021). In the endoplasmic reticulum, glycosylation is crucial for protein folding, stability, and function. In glycosylation, N-glycan attachment to fungal proteins modifies the effectiveness of the pathogen-host cell relationship. In contrast to the mammalian complex and hybrid forms of N-glycans, which also contain other sugars in addition to mannose, the majority of the N-glycans of yeast glycoproteins have a high mannose content. A mannoprotein holds the N-glycan in place in the fungus Cryptococcus. A protein found in the polysaccharide capsule is called mannoprotein. Particularly MP98, mannoproteins comprise 455 amino acids as well as chitin deacetylases in the 152nd–274th domain. The protein MP98 is linked to GPI at the C-terminal omega site and has an N-terminal signal sequence that has been cut. The asparagine-associated glycosylation pathway (ALG) genes encode a group of specialized glycosyltransferases that are found in the ER and are responsible for assembling the N-glycan core in eukaryotic cells (Lee et al., 2023). According to a study, the core of the N-glycan is crucial to Cryptococcus neoformans’ ability to cause host cell death. This study made use of strains that have ALG3, ALG9, and ALG12 deletions. These three genes generate mannosyl transferases that attach to lipids to form the nucleus of N-glycans, and they are dependent on the lipid Dol-P-Man mutant When compared to the non-mutant strain, the mutant strain’s loss of the fungal cell wall resulted in a lower rate of macrophage cell death. These findings suggest that the control of cell death, which can be a dissemination evasion tactic, is influenced by the N-glycan structure (Thak et al., 2020).

### Extracellular Molecules

Cryptococcus secreted some types of proteins, there are Phospholipase, Urease, Protease, and Cpl1. Phospholipase enzymes are a varied class of phospholipid-cleaving enzymes that produce a wide range of chemical substances with physiologically active properties. The fluctuating environments that allow *Cryptococcus neoformans* to exist in the host are affected if the phospholipids are broken down. In addition, the activity of phospholipases results in membrane instability and host cell lysis. Another role of phospholipase is to promote endocytosis during BBB crossing (Evans et al., 2015; Chen, Y., Shi, Zoe W, *et al.*, 2022; Hamed et al., 2022).

Two other enzymes implicated in the evasion system are protease and urease. Host proteins like collagen, elastin, fibrinogen, immunoglobulins, and complement are broken down by protease activity, whereas urease catalyzes the breakdown of urea into ammonia and carbonate. Endothelial cells may get damaged and barrier permeability may increase as a result of the extracellular enzymatic breakdown of urea to dangerous ammonia (Almeida, Julie M Wolf and Casadevall, 2015; Yang, Wang and Zou, 2017; Chen, Y., Shi, Zoe W, et al., 2022).

One more protein, Called Cpl1, is secreted by Cryptococcus neoformans. Through the induction of TLR-4 signaling, type 2 immunity can be activated by Cpl1, which also causes macrophages to produce arginase-1. The IL-4 signaling pathway and STAT3 phosphorylation can be enhanced by macrophages’ arginase activation. Cpl1 stimulates type-2 immunity during a cryptococcal infection by activating macrophages. A favorable environment for Cryptococcus neoformans development can be created by type 2 immunity (Bordon, 2022; Chen, Y., Shi, Zoe W, et al, 2022; Dang et al., 2022; León, 2022).

In addition, Small compounds secreted by Cryptococcus are immunologically active and affect IL-1β inflammasome-dependent secretion, but the mechanism of action and signaling pathway unclearly. Recently in a study using murine BDMC and BMMs samples infected with CM35. CM 35 was generated from yeast culture strain B3501, which was cultured in media for five days before being centrifuged to remove yeast cells. The supernatant was collected and filtered. CM35 is fractionated at several sizes, including < 1 kDa. In addition, CM35 was autoclaved and treated with proteases such as trypsin, thermolysin, and pronase before being processed to remove GXM. The results showed that <1 kDA had the greatest effect on decreasing IL-1 because it decreased the action of caspase-1, which cleavages pro-IL-1β. Furthermore, these investigations indicated that < 1 kDa CM35 affects macrophage function to influence antifungal activity, perhaps through the production of chemicals that suppress the pro-inflammatory milieu, so influencing pathogenesis. (Bügel et al., 2020).

### Antioxidant systems: Prx, Grx, Trx, catalase, and SOD system

Melanin is a hydrophobic pigment with a negative charge that is found in the cell wall of Cryptococcus (Chatterjee et al., 2015). Melanin protects fungi from UV radiation and severe temperatures in nature, and its antioxidant qualities can protect fungi against ROS produced by macrophages in the host(Cordero et al., 2020). Furthermore, melanin can block the release of IL-12, IL-1, TNF-α, IFN-γ, and iNOS. Laccase is an enzyme that is used in the biosynthesis of melanin through L-DOVA as a substrate (Barluzzi et al., 2000; Qiu et al., 2012; Almeida, Julie M. Wolf and Casadevall, 2015). Melanogenesis begins by activation of Gsk3 kinase. Gsk kinase induces melanin-regulating core transcription factors (MRC-TFs): Hob1, Bzp4, Usv101, and Mbs1 via Lac1 expression under low-nutrient conditions and the presence of melanin precursor L-DOVA. Each transcription factor induces Lac1 expression and modulates epistatic interaction between c-AMP, RAM, and HOG pathways. In addition, MRC-TFs also regulate the transport and aggregation of melanin in the fungal cell wall. Meanwhile, the Product of Melanin expression transports using The Vsp15-Vsp30-Vsp34 complex, On another side the mannoprotein Cig1 interact with melanin and function as iron homeostasis in fungal cells ( Cordero et al., 2020).

Cryptococcus can evade the immune response by releasing compounds that can inhibit oxidative mechanisms, such as the Prx, Trx, Grx/GSH, catalase, and SOD systems. The Prx system, also known as a thiol oxidase, is a 20-30 kDA molecule that protects against free radicals by eliminating peroxides. Trx, which comprises NADPH, Trx, and Thioredoxin reductase (TrxR), is located downstream of Prx and is involved in the regulation of DNA synthesis, transcription, cell proliferation, and apoptosis. Trx is a dithiol oxidoreductase that acts as the primary carrier of redox potential in cells, as a cofactor for important enzymes, and as a protein repair enzyme via methionine sulfoxide reductase. It also reduces protein disulfide. The Trx system’s redox regulation controls the expression of numerous enzymes involved in stress defense and protects cells from oxidative stress (Yang, Wang and Zou, 2017).

The *Cryptococcus neoformans* Grx or GSH system is a thiol-dependent antioxidant system. Cryptococcus is vulnerable to oxidative components such as peroxides, superoxide anions, and hazardous products of lipid peroxidation due to GSH deficiency. GRX is encoded by the GRX1 and GRX2 genes in Cryptococcus neoformans. Cryptococcus which has lost its GRX1 gene may be vulnerable to oxidative stress generated by superoxide anions, whereas Cryptococcus which has lost its GRX2 gene may be sensitive to oxidative stress induced by hydrogen peroxide. Furthermore, GSH or Grxs can cleave organic hydroperoxides because GSH works as a reducing agent or in hazardous lipophilic conjugation (Yang, Wang and Zou, 2017; Wangsanut and Pongpom, 2022).

Catalase enzymes, also known as antioxidant metalloenzymes, cleaved hydrogen peroxide to form H_2_O and O_2_, and SOD is classified as Cu/Zn-SOD (also known as SOD1) and Mn-SOD. (Known as SOD2). SOD1 functions in the evasion system as an antioxidant defense against ROS, whereas SOD2 is essential for cellular antioxidants against superoxide stress (Yang, Wang and Zou, 2017; Aaron D. Smith et al., 2021; Kumari et al., 2021).

### Molecular Interaction of Cryptococcus with Blood-Brain Barrier

If Cryptococcus escapes against the immune system, Cryptococcus will disseminate from the lungs to other organs, including the central nervous system, where it can penetrate the blood-brain barrier. Brain microvascular endothelial cells (BMEC), pericytes, astrocytes, and the basement membrane (BM) make up the blood-brain barrier. (Xu, Nirwane and Yao, 2019). Endothelial cells border the lumen side of the brain vasculature and are linked together by tight junctions. During inflammation, circulating leukocytes can enter the brain parenchyma either paracellularly via the tight junction or transcellularly by endocytosis mediated by endothelial cells.(Kourtzelis et al., 2017; Lochhead et al., 2020). This fungus enters the BBB using the following mechanism:

### Trans-cellular crossing

Cryptococcus HA binds to the CD44 receptor and activates EpHA2. EpHA2 promotes activation of the Rho-GTPase signaling pathway and induces cytoskeletal remodeling for internalization. Research has shown that blocking her EphA2 activity with antibodies or chemical inhibitors prevented the transmigration of C. neoformans in a BBB in vitro model (Aaron et al., 2018). On the other hand, Cryptococcus secretes Mpr1 to promote endothelial cell adhesion. Studies have shown that the Mpr1 strain *Cryptococcus neoformans*, which lacks the gene encoding Mpr1 failed to penetrate the endothelium in a BBB in vitro model (Vu et al., 2014). In addition, Cryptococcus also secretes her PLB-1, which subsequently activates rac1 and triggers endocytosis, allowing the fungus to enter the cell. After successful internalization, the fungus requires exocytosis through the interaction of her AnX2 and Mpr1(Strickland and Shi, 2021; Chen, Y., Shi, Zoe W, et al., 2022).

### Trojan Horse

Trojan-horse occurs in HIV-Patient because the barrier permeability is weakened so that monocytes more easily pass through the blood barrier than Health. Cryptococcus uses macrophages as transporters to reach blood vessels. Macrophages migrate through the blood vessels along with the Cryptococcus inside. In vitro hCMEC studies showed that C. neoformans-infected monocytes directly crossed the endothelium and promoted neural invasion (Sorrell et al., 2016; 35. Santiago-Tirado, F. H. et al., 2017). Another study showed that monocytes were recruited on CXCR3+Ly6C^low^ 12 hours after *Cryptococcus neoformans* infection. This result indicates that cryptococcal-engulfed monocytes adhere to the luminal walls of cerebral microvessels and migrate into the parenchyma.(Sun et al., 2020)

###  Paracellular

In paracellular mechanism, Cryptococcus produces Ure1, mannoprotein, and protease to break tight junctions. Ure1 can damage cell junction proteins by converting urea to toxic ammonia, and plasminogen is converted to serine protease plasmin, and proteases interfere with the BBB. These three proteins are secreted to damage the tight junction so Cryptococcus can successfully pass through the BBB.(Sabiiti and May, 2012; Chen, Y., Shi, Zoe W, et al, 2022).

**Figure 2:**
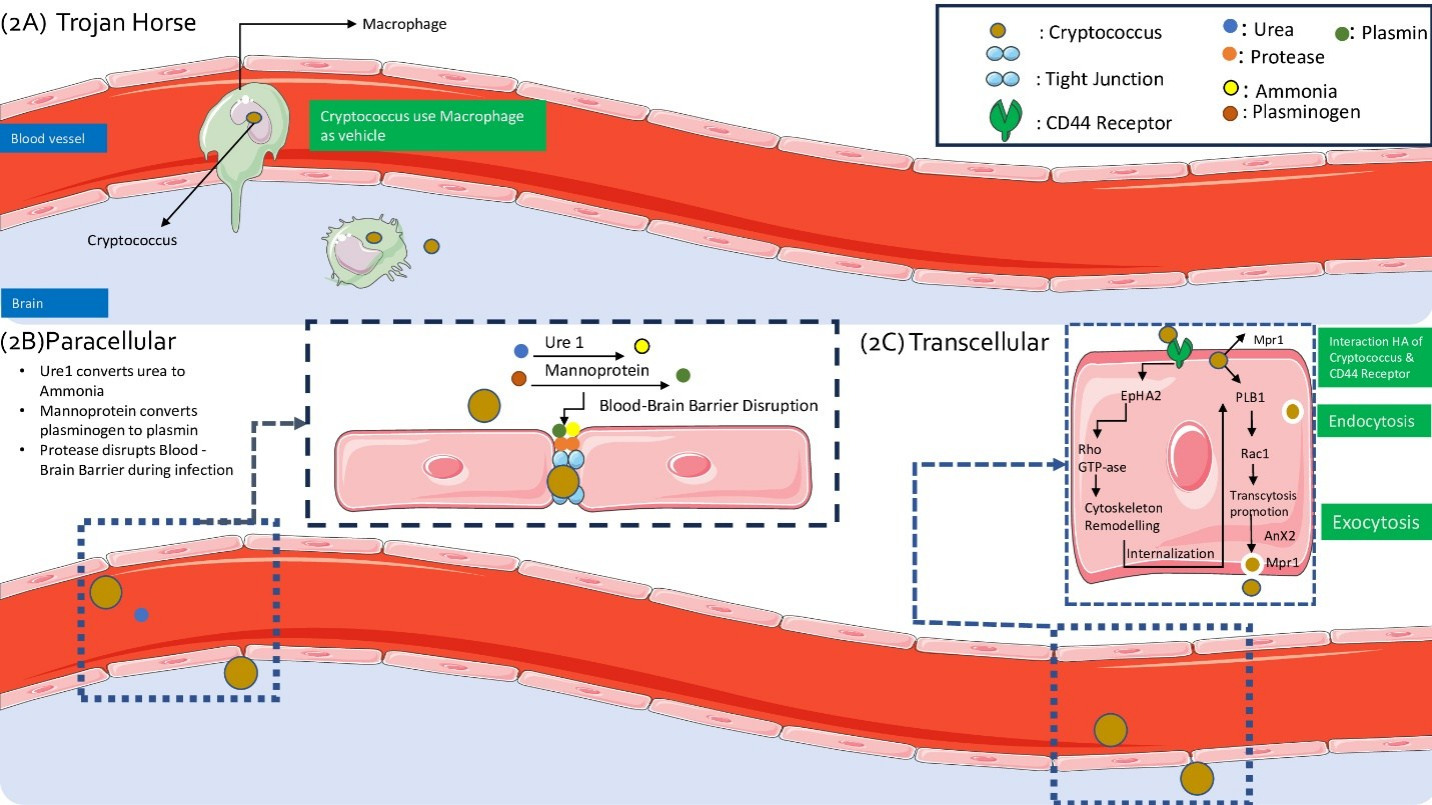
Biological interaction of Cryptococcus with endothelial cells. Figure 2A =Trojan Horse; Figure 2B = Transcellular crossing; and Figure 2C = Paracellular mechanisms. Parts of the figure were drawn using pictures from Servier Medical Art. Servier Medical Art by Servier is licensed under a Creative Commons Attribution 3.0 Unported License (https://creativecommons.org/licenses/by/3.0/)

In addition to the three mechanisms above, recent studies have shown that when an HIV attack occurs, the Cryptococcus fungus synergies with HIV crosses the blood-brain barrier via CD44(Chen, L et al., 2020). HIV contains gp120 and gp41-I90 regions that are linked to Cryptococcus via CD44. The results demonstrated that Cryptococcus infection boosted CD44 expression in HBME by collaborating with gp120. This showed that HIV-gp 120 and CD44 interacted synergistically to promote Cryptococcus invasion of HBME in vitro(Cao et al., 2020). Other research has found an association between gp41-I90 and Cryptococcus in crossing the BBB via CD44 in monocytes. The findings suggested that HIV-gp41-I90 facilitated monocyte transmigration across the BBB by CD44 infection. Cn-mediated leukocyte migration is regulated by CD44. Cn infection of BMEC boosted monocyte cell adherence and motility across the monolayer considerably(He et al., 2016; Huang et al., 2022).

## Conclusion

Cryptococcosis is a systemic mycotic disease caused mostly by *Cryptococcus neoformans*. Basidiospores enter alveoli and will be phagocytized by macrophages alveolar. In persons with healthy immune systems, cryptococcal infections are uncommon; nevertheless, in immunocompromised patients, Cryptococcus can activate the evasion system and down-regulate the immune response, and the fungus dissemination to other organs, such as the brain, to cause damage.
